# A novel virus potentially evolved from the N4-like viruses represents a unique viral family: *Poorviridae*

**DOI:** 10.1128/aem.01559-24

**Published:** 2024-11-21

**Authors:** Wei Wang, Hongmin Wang, Xiao Zou, Yundan Liu, Kaiyang Zheng, Xin Chen, Xinyi Wang, Shujuan Sun, Yang Yang, Min Wang, Hongbing Shao, Yantao Liang

**Affiliations:** 1 College of Marine Life Sciences, MoE Key Laboratory of Evolution & Marine Biodiversity, Institute of Evolution and Marine Biodiversity, Frontiers Science Center for Deep Ocean Multispheres and Earth System, Center for Ocean Carbon Neutrality, Ocean University of China, Qingdao, China; 2 Xiangdong Hospital, Hunan Normal University, Liling, China; 3 UMT-OUC Joint Centre for Marine Studies, Qingdao, China; 4 Haide College, Ocean University of China, Qingdao, China; 5 The Affiliated Hospital of Qingdao University, Qingdao, China; Colorado School of Mines, Golden, Colorado, USA

**Keywords:** *Pseudoalteromonas*, virus, genomic diversity, N4-like virus, evolution

## Abstract

*Pseudoalteromonas* are widely distributed in marine extreme habitats and exhibit diverse extracellular protease activity, which is essential for marine biogeochemical cycles. However, our understanding of viruses that infect *Pseudoalteromonas* remains limited. This study isolated a virus infecting *Pseudoalteromonas nigrifaciens* from Xiaogang in Qingdao, China. vB_PunP_Y3 comprises a linear, double-strand DNA genome with a length of 48,854 bp, encoding 52 putative open reading frames. Transmission electron microscopy demonstrates the short-tailed morphology of vB_PunP_Y3. Phylogenetic and genome-content-based analysis indicate that vB_PunP_Y3 represents a novel virus family named as *Poorviridae*, along with three high-quality uncultivated viral genomes. Biogeographical analyses show that *Poorviridae* is distributed across five viral ecological zones, and is predominantly detected in the Antarctic, Arctic, and bathypelagic zones. Comparative genomics analyses identified three of the seven hallmark proteins of N4-like viruses (DNA polymerase, major capsid protein, and virion-encapsulated RNA polymerase) from vB_PunP_Y3, combing with the protein tertiary structures of the major capsid protein, suggesting that vB_PunP_Y3 might evolve from the N4-like viruses.

**IMPORTANCE:**

vB_PunP_Y3 is a unique strain containing three of the seven hallmark proteins of N4-like viruses, but is grouped into a novel family-level viral cluster with three uncultured viruses from metagenomics, named *Poorviridae*. This study enhanced the understanding about the genetic diversity, evolution, and distribution of *Pseudoalteromonas* viruses and provided insights into the novel evolution mechanism of marine viruses.

## INTRODUCTION

Viruses widely distributed in the ocean, are considered the richest genetic reservoir on Earth (1). Among these, the most noteworthy are viruses infecting bacteria (2). Although opinions differ on the impact of viruses on the oceanic carbon sink, it is undeniable that viruses significantly influence global marine ecosystems. Their effects include alterations in population size and diversity, the transfer of genetic material between organisms, and the recycling of nutrients and organic carbon through the lysis of planktonic organisms (3, 4). However, the majority of virus sequences compiled from metagenomic data are difficult to align with any recognized nucleotide or amino acid sequences, categorized as “viral dark matter” (5). The discovery of new strains can greatly reduce the amount of “viral dark matter.” Thus, the combination of isolating and sequencing of marine viruses with metagenomic methodologies becomes particularly crucial.

*Pseudoalteromonas*, a representative genus in the *Pseudoalteromonadaceae* family, is widely distributed across diverse marine environments, especially in extreme habitats like deep-sea and polar regions (6). Although its presence may not be notably high in surface waters, it frequently accumulates on suspended particles in these areas, and emerges as a dominant bacterial group in deep-sea settings (7–10). This vertical distribution highlights the evolutionary adaptation of the species to its surroundings and its potential impact on vertical carbon cycling within the marine ecosystem (11, 12). Furthermore, it possesses the capacity to produce many biologically active natural compounds, such as antimicrobial agents, antifouling substances, and algicidal compounds (13–15). However, the understanding of viruses infecting *Pseudoalteromonas* remains limited. As of 2 September 2024, among the 28,825 isolated viruses documented in the GenBank database, only 48 strains have the ability to infect *Pseudoalteromonas*.

N4-like viruses, recently classified into the novel viral family *Schitoviridae* in 2021, are part of a podoviral-like lineage and it is characterized by seven hallmark proteins, including DNA polymerase (DNAP), major capsid protein (MCP), portal protein, tail protein, terminase large subunit (TerL), terminase small subunit (TerS), and virion-encapsulated RNA polymerase (RNAP) (16). They exhibit conserved genomic characteristics and a unique replication mechanism (17, 18). These viruses can lytically infect many pathogenic bacteria, displaying efficient therapeutic performance in previous animal experiments (19–24). This makes them promising candidates for virus therapy against multidrug-resistant pathogenic bacteria.

Here, a novel virus was isolated and identified, vB_PunP_Y3 potentially evolved from the N4-like viruses, infecting *Pseudoalteromonas nigrifaciens*. Not only its morphological, physiological, and genomic characteristics were described, genomic analysis and comparative genomics analysis were conducted. This provides a crucial foundation for further exploration of the adaptive evolution and ecological roles of marine viruses and their hosts in ocean.

## MATERIALS AND METHODS

### Isolation and purification

The vB_PunP_Y3 and its host were collected from Xiaogang in Qingdao, China on 4 September 2021 (36.1000°N, 120.4833°E; *T* = 24.7℃; pH = 8.38; SAL = 27.33 ppt; DO = 8.66 mg/L). The sample water was diluted to 10^−4^ in a stepwise manner, and 200 µL of the diluted sample was inoculated onto 2216E solid medium using the plate coating method (25). The sample was incubated at 28°C, and then the host bacteria were purified four times. The 16S rRNA of the purified host was amplified using PCR and sequenced. The host was identified through BLASTn search and phylogenetic analysis based on the 16S rRNA sequence (26).

The double-layer plate method was used in viral isolation (26). Sampled seawater was filtered through 0.22 µm pore-size membranes as virion suspension. A suspension was made by mixing 200 µL of sample filtrate and 200 µL of the host culture in the logarithmic growth phase in a cryopreservation tube and left to stand at 25°C for 25 min. The mixture was then injected into 4.5 mL of semisolid medium melted at 55°C and poured onto the surface of the solid medium. After culturing the agar plate in an incubator at 28℃ for 24 h, the formation of plaques was observed. If there was a plaque on the plate, it was picked out and placed in 1 mL of SM Buffer (100 mM NaCl, 8 mM MgSO_4_⋅7H_2_O, 50 mM Tris-Cl, pH = 7.5), and then filtered into a 0.22 µm PES Millipore filter. After the filtrate was gradually diluted, the infection step was repeated three times to ensure that the virus solution was completely purified (27).

The purified virion suspension was used to infect the host culture. The 4 L lysis solution was treated with 10% (wt/vol) PEG6000 at 4°C for 12 h to obtain concentrated virions. The PEG6000 treated lysis solution was centrifuged (4,500 × *g*) for 30 min at 4°C. The supernatant was then gently poured out without disturbing the deposit (28). The precipitate was resuspended in 4 mL SM buffer to obtain concentrated virions, which were stored in SM buffer at 4°C.

### Transmission electron microscopy

The viral morphology was analyzed using transmission electron microscopy (TEM). Purified virus particles were negatively stained with 1% wt/vol phosphotungstic acid at pH 7.0 (29). Observations were made using a JEOL Model JEM-1200EX TEM operating at 100 kV. Virus dimensions were estimated from the electron micrographs (30).

### One-step growth curve of vB_PunP_Y3

The double-layer plate method was employed to determine the one-step growth curve. The virion suspension of vB_PunP_Y3 was combined with the host culture in the logarithmic growth phase at a multiplicity of infection (MOI) of 0.1, and incubation at 28°C (31). To enhance the attachment of viruses to the host bacteria, the suspensions were incubated for 20 min before subsequent centrifugation. Following this, the infected culture was centrifuged at 8,000 × *g* for 1 min, and the supernatant was discarded to remove unabsorbed viruses. The pellet was then resuspended in 1 mL of 2216E liquid medium, centrifuged again at 8,000 × *g* for 1 min, and the supernatant was discarded. After repeating this process three times, the cells were resuspended in 50 mL of fresh 2216E liquid medium. Samples were collected at 5 min intervals (up to 30 min), 10 min intervals (up to 90 min), and 30 min intervals (up to 180 min), after which the virus titer was determined through plaque assays (32).

### Temperature, pH, and UV stability assays

Samples with a titer of approximately 10^8^ PFU/mL (pH = 7.0) were collected, and 2 h at different temperatures, including −20°C, 4°C, 25°C, 35°C, 45°C, 55°C, 65°C, and 75°C. Three replicates were established for each temperature condition. The virus solution treated under each temperature condition was combined with the host bacterial culture in the exponential growth phase. After an incubation period of 20–30 min, the resulting mixture was poured onto double-layer agar plates and left to incubate for 12 h. Subsequently, the virus titer was determined for each temperature condition, and a trend graph was created to illustrate the impact of temperature fluctuations on virus growth.

A 5 mL volume of virus solution with an initial titer of 10^8^ PFU/mL was prepared and its pH was adjusted using HCl and NaOH. Subsequently, 11 aliquots of the virus solution were prepared, covering a pH range from 2 to 12. The virus solution was then incubated at 28℃ for 2 h. Following this, 200 µL of the virus solution at each pH level was mixed with an equal volume of host bacterial solution, which was in the logarithmic growth phase and had a concentration of 10^7^ CFU/mL. The resulting blends are processed by the double-layer agar plate method. Ultimately, the quantity of virus plaques on the agar plates was counted, and a trend graph was generated to depict the impact of distinct pH conditions on virus proliferation (33).

Virus solution with an approximate titer of 10^8^ PFU/mL was exposed to a 20 W ultraviolet (UV) lamp (34). Sampling was initiated at 10 min intervals and extended to 30 min intervals after 60 min. In each instance, the quantity of virus plaques on the agar plates was assessed through the double-layer plate method. Ultimately, a graph was constructed to depict the influence of UV irradiation duration on the sample titer.

### Viral genome sequencing and annotation

The Virus DNA Kit (OMEGA) was utilized to perform DNA extraction of vB_PunP_Y3 (31). Viral genomic next-generation sequencing (NGS) was powered by BioZeron Company in Shanghai, China. Raw data were assembled by the software ABySS (v1.3.7) (35) to obtain initial genomic fragments, whose gaps were filled by GapClose (v1.12) software with default parameters. Viral genome assembled completeness (occurrence of directed terminal repeat) is assessed by CheckV (v1.0.1) (36). Additionally, no sequences with over 90% similarity to vB_PunP_Y3 were found in the National Center for Biotechnology Information (NCBI) database.

Open reading frames (ORFs) were predicted through the utilization of a combination of PRODIGAL (v2.6.3), RAST (https://rast.nmpdr.org/rast.cgi), and GeneMarkS (http://topaz.gatech.edu/GeneMark/) (37). The initiation codon and termination codon of each ORF were manually scrutinized to prevent any disruption in the case of lengthy protein sequences. The sequences of the ORFs were then aligned with GenBank’s non-redundant (nr) protein database (https://blast.ncbi.nlm.nih.gov/), the Pfam database (38), KEGG Orthologs (KO) database (39), and the HHpred database (40), *e*-value was set at <1e−5. All comparison results were manually corrected. The genome organization diagram was generated using CLC Main Workbench 20. Additionally, tRNA prediction was carried out using the tRNAscan-SE program (http://lowelab.ucsc.edu/tRNAscan-SE/) (41). The GC-skew was calculated and visualized by Genskew (https://genskew.csb.univie.ac.at/webskew) (42). A window size and step of 100 bp were employed to calculate the global minimum and maximum, displayed in the cumulative graph.

### Tetranucleotides correlations analysis

Nineteen segments were extracted from the genomes of vB_PunP_Y3 (window size: 10 kb, step size: 2.5 kb). The genomes were initially concatenated to preserve their integrity, such that each 2.5 kb segment of the genomes corresponded to a respective 10 kb fragment (43). For each segment, 256 potential combinations of tetranucleotide frequency (from “AAAA” to “TTTT”) were computed and standardized using the z-scoring algorithm. Pearson’s correlation coefficients (*R*-values) were computed from an array of *z*-scores in comparison with the entire genome of the virus (44).

### Genome network analysis of vB_PunP_Y3

Uncultivated viral genomes (UViGs) closely related to vB_PunP_Y3 were mined from the IMG/VR (v.4) data set by BLASTp algorithm-based analysis. The analysis parameters were set to an *E*-value of less than 1e−5 and at least 30% protein sharing between the target genomes and vB_PunP_Y3 (26). A total of 12,520 genomes with taxonomic status were downloaded from International Committee on Taxonomy of Viruses (ICTV), as reference genomes, and were merged with UViGs closely related to vB_PunP_Y3. Genome-wide redundancy was eliminated using CD-HIT (v4.8.1) with parameters (-c 0.99 -aL 0.9 -aS 0.9s 0.99) (45). All-to-all BLASTp (*E*-value <1e−5) was employed on all proteins using DIAMOND (2.1.6). Proteins were clustered using the Markov clustering algorithm (MCL) to generate protein clusters (PCs). The weights between different genomes were described by similarity scores calculated by vConTACT (v2.0). Viral clusters (VCs) were assigned using ClusterONE (46). All genomes with weights related to vB_PunP_Y3 were selected for network analysis and visualized using Gephi (v0.9.7).

### Phylogenetic and comparative genomic analysis

The circular proteomic tree of the VC containing vB_PunP_Y3 was generated based on whole-genome sequence similarity using ViPTree (https://www.genome.jp/viptree/) (47). Subsequently, the branch containing vB_PunP_Y3 was separately displayed by drawing a rectangular proteomic tree. The average nucleotide identity (ANI) can be used to show the homogenetic relationship at the nucleotide level, and ANI values were computed to evaluate genetic relatedness among members of the VC containing vB_PunP_Y3, utilizing the Virus Intergenomic Distance Calculator (VIRIDIC) (48, 49). The phylogenetic tree of TerL was calculated by IQ-Tree2 (v2.0.3) (50). Homologous protein analysis was conducted for vB_PunP_Y3 and related reference genome, based on the protein clustering results. Genomic comparison analysis of members of the candidate family (IMGVR_UViG_2579778892_000013, IMGVR_UViG_3300039449_000044, IMGVR_UViG_3300037572_000254, and vB_PunP_Y3) was conducted using tBLASTx.

### The ecological footprint of *Poorviridae*

The Global Ocean Viromes 2.0 (GOV2.0) data set provides insight into the biogeographical distribution of *Poorviridae* in marine environments, which have been divided into five viral ecological zones (VEZs), including Antarctic (ANT), Arctic (ARC), temperate and tropical epipelagic (EPI), bathypelagic (BATHY), and tropical mesopelagic (MES) (51). The relative abundance of *Poorviridae* was expressed as TPM (transcripts per million mapped reads) and calculated using Cover (v0.3.0) with parameters: 95% minimum reads identity and 75% minimum reads alignment. The values were normalized by the number of databases in each VEZ and transformed by log_10_(*X* + 1). The reads from the 154 data sets of GOV2.0 were mapped to the *Poorviridae* genome by minimap2 (v2.17). The representative marine viruses that exhibited high relative abundance in the global oceans were used as references, including the cyanoviruses (P-SSP7, P-SSM7, S-SSM7, and S-SM2), pelagiviruses (HTVC010P, HTVC011P, and HTVC019P), and other 14 viruses with high relative abundance (52, 53).

### Comparative genomic analysis between vB_PunP_Y3 and N4-like Viruses

All UViGs are quality assessed using CheckV (v1.0.1) (36), and only sequences classified as complete and high-quality are retained (16). Finally, 148 non-redundant high-quality UViGs, 154 viral isolates, and 40 integrated proviruses from an existing data set of N4-like viruses were integrated into the reference database and compared to vB_PunP_Y3 using all-to-all Position-Specific Iterated BLAST (PSI-BLAST), which identifies distant relatives for a protein family by iteratively searching position-specific score matrix (PSSM) (https://ftp.ncbi.nlm.nih.gov/pub/factsheets/HowTo_BLASTGuide.pdf). Subsequently, proteomic tree of the reference database and vB_PunP_Y3 was generated based on whole-genome sequence by ViPTree and visualized by iTol (v5.0), and the phylogenetic trees were calculated using IQ-Tree2 (v2.0.3) based on the amino acid sequences of DNAP, MCP, and RNAP. Additionally, comparing N4-like viruses and vB_PunP_Y3 from the aspects of the protein tertiary structure of MCP and morphology. ESMFold (54) was employed for protein tertiary structure prediction, and RCSB Protein Data Bank (RCSB-PDB) (https://www.rcsb.org/) was utilized for protein visualization and comparison.

## RESULTS AND DISCUSSION

### Identification of the host bacteria

BLASTn search indicated that the 16S rRNA nucleotide sequence similarity between the host bacteria and *P. nigrifaciens* is 99.51%. Phylogenetic analysis of the 16S rRNA sequence showed that host bacteria and *P. nigrifaciens* are in the same branch (Fig. S1).

### Morphology and characterization of vB_PunP_Y3

**Fig 1:**
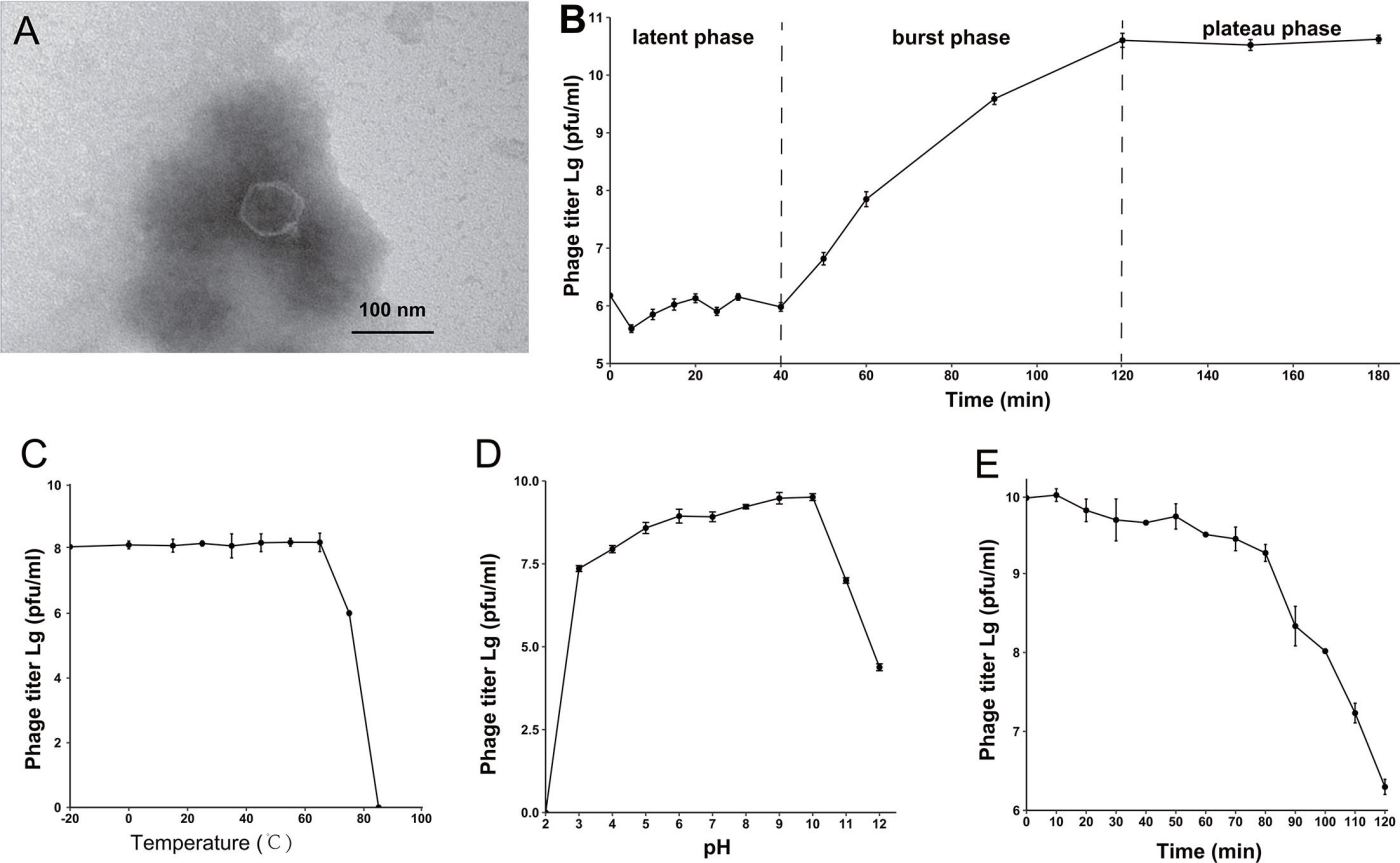
Morphology and physiology of phage vB_PunP_Y3. (A–E) Morphology, one-step growth curve, temperature stability, pH stability, and UV stability of phage vB_PunP_Y3, respectively.

The result of TEM demonstrated that the virus vB_PunP_Y3 has an icosahedral head with a diameter of approximately 70 nm and a non-contractile tail of about 20 nm (Fig. 1A). The one-step growth curve revealed an extended latent phase (0–40 min) and a burst phase (40–120 min), with subsequent stability in the viral plaque forming unit (PFU) over time (Fig. 1B). The burst size of phage vB_PunP_Y3 was calculated to be approximately 67 viral particles per cell. Notably, vB_PunP_Y3 has extreme temperature stability, maintaining consistently high PFU from −20°C to 65°C. Above 75°C, its viability decreases, and at 85°C, all biological activities were lost (Fig. 1C). vB_PunP_Y3 is well adapted to a wide pH range (pH = 2–12), but begins to decline in biological activity at pH = 9. This suggests that vB_PunP_Y3 is resistant in alkaline environments (Fig. 1D). Exposed to UV radiation, the PFU gradually decreased over 0–120 min. However, even after 120 min, vB_PunP_Y3 retained considerable biological activity, indicating its significant resistance to UV radiation (Fig. 1E).

### Genomic characteristics of vB_PunP_Y3

**Fig 2:**
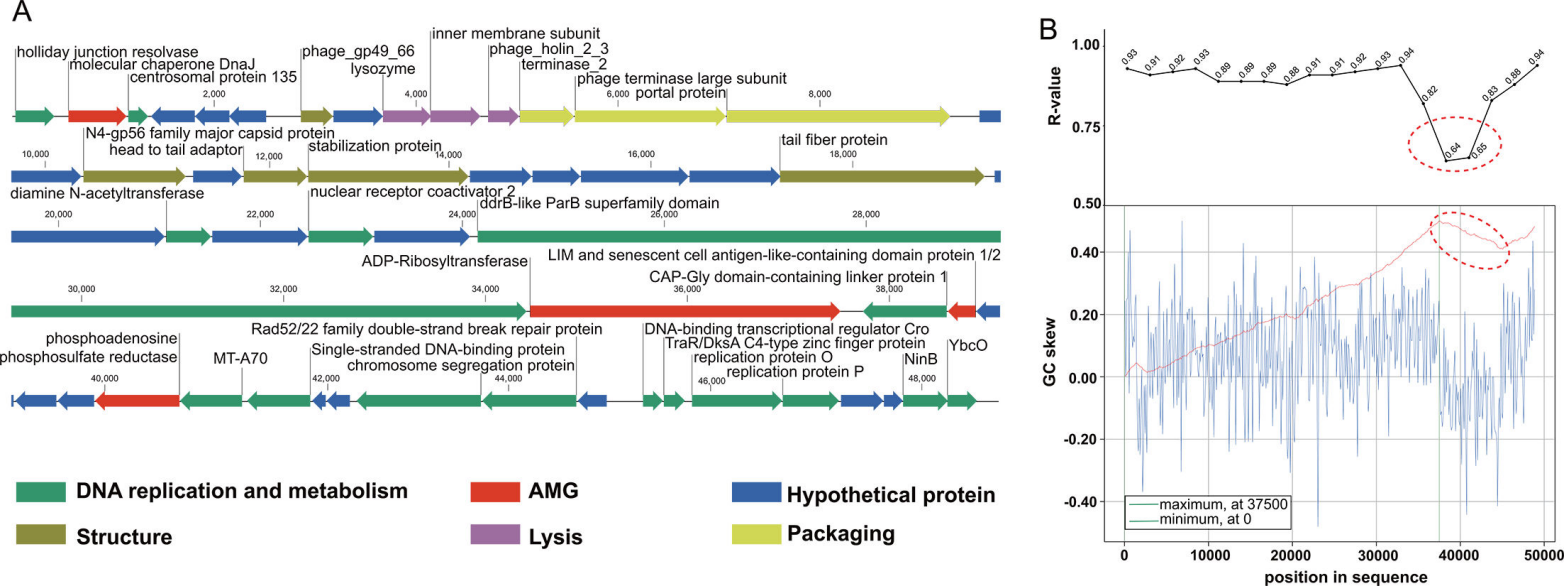
Genomic analysis of phage vB_PunP_Y3. (A) Genome map of vB_PunP_Y3, with functional categories indicated by color. Arrow length represents ORF size, color denotes ORF classification, and arrow direction shows strand orientation. (B) Tetranucleotide correlation and GC-skew analysis. Top: Tetranucleotide correlation (window size: 10 kb, step size: 2.5 kb), with lower R-value highlighted in red ellipse. Bottom: Cumulative GC-skew analysis, with GC-skew and cumulative GC-skew shown as blue and red lines, respectively; decreasing cumulative GC-skew is highlighted in red ellipse.

The genome of vB_PunP_Y3 consists of a linear, double-stranded 48,854 bp DNA molecule with a G+C content of 45.4%; no tRNA genes were annotated (Fig. 2A). The coding ratio of the virus genome is 95.95%. The prediction results showed the presence of 52 ORFs within the genome. Among these, 36 ORFs were detected in the positive strand, while the remaining 16 were located on the negative strand. The majority of ORFs (*n* = 49) were found to be initiated by the codon ATG, whereas three were initiated by the alternative codon GTG. According to the ORFs annotations, 21 ORFs, approximately 40% of the total, had unknown functionalities and were classified as hypothetical proteins. Furthermore, 31 ORFs were aligned with various functions, including auxiliary metabolic genes (AMGs, *n* = 4), structure proteins (*n* = 5), packaging proteins (*n* = 3), DNA replication and metabolism proteins (*n* = 16), and lysis proteins (*n* = 3).

The genome of the virus vB_PunP_Y3 contains three genes related to lysis named lysozyme, inner membrane subunit and Phage_holin_2_3. Lysozyme, an antimicrobial enzyme part of the innate immune system, hydrolyzes (1–4)-β-linkages in peptidoglycans, affecting the cell walls of gram-positive bacteria (55). Abundant in secretions and eggs (56), it displays thermal stability (57), varied pH activity (58), and acts on chitin as well (59). Component of the spanin complex that disrupts the host outer membrane and participates in cell lysis during virus exit. The spanin complex conducts the final step in host lysis by disrupting the outer membrane after holin and endolysin action have permeabilized the inner membrane and degraded the host peptidoglycans. Host outer membrane disruption is possibly due to local fusion between the inner and outer membrane performed by the spanin complex (by similarity) (60). Phage_holin_2_3 is a family of small hydrophobic virus proteins called holins with one transmembrane domain. Holins are produced by double-stranded DNA viruses that use an endolysin-holin strategy to achieve lysis of their hosts. Endolysins are peptidoglycan-degrading enzymes that are usually accumulated in the cytosol until access to the cell wall substrate is provided by the holin membrane lesion (61).

Additionally, within the genome of virus vB_PunP_Y3, four AMGs were identified: the molecular chaperone DnaJ (ORF2), ADP-Ribosyltransferase (ORF31), LIM and senescent cell antigen-like-containing domain protein 1/2 (LIM-SC1/2) (ORF33), and phosphoadenosine phosphosulfate reductase (ORF37). Chaperone DnaJ, known as Hsp40, is a molecular chaperone found across diverse organisms from bacteria to humans (62, 63). It safeguards proteins from aggregating irreversibly during synthesis and cellular stress. A family of ADP-ribosyltransferases has been identified in polyvalent proteins of bacteriophages and conjugative elements. These enzymes are associated with the Tox-ART-HYD2 group of ADP-ribosyltransferases, which are commonly found in polymorphic toxin systems and toxin-antitoxin systems. It is predicted that these enzymes modify host proteins (64). LIM-SC1/2 contains LIM domains that facilitate protein-protein interactions. These proteins interact with integrin-linked kinase (ILK) and likely function as adaptor proteins, linking ILK with other signaling components involved in cell adhesion and growth factor signaling. They may also play a role in cellular aging processes (65, 66). Phosphoadenosine phosphosulfate reductase catalyzes the reduction of phosphoadenosine phosphosulfate to sulfide, aiding sulfur assimilation for the synthesis of sulfur-containing amino acids. This function is crucial for survival in anoxic environments (67).

The tetranucleotide correlation between the entire genome of vB_PunP_Y3 and each 10 kb genome segment is illustrated in Fig. 2B. With the exception of genes circled by the red ellipse, all other genes exhibit Pearson’s correlation coefficients higher than 0.9 with the entire genome, indicating a strong association between these genes and the complete genome of vB_PunP_Y3. The results of the cumulative GC-skew analysis showed a similar trend to the tetranucleotide correlation, with a decreasing trend observed in the same region (Fig. 2B). Furthermore, within the area circled by the red ellipse, three AMGs of the virus vB_PunP_Y3 are distributed: ADP-Ribosyltransferase (ORF31), LIM-SC1/2 (ORF33), and phosphoadenosine phosphosulfate reductase (ORF37). It is likely that the presence of these AMGs leads to a decrease in the values of cumulative GC-skew analysis and a reduction in the strength of tetranucleotide correlation.

### Virus vB_PunP_Y3 represents a new family: *Poorviridae*

**Fig 3:**
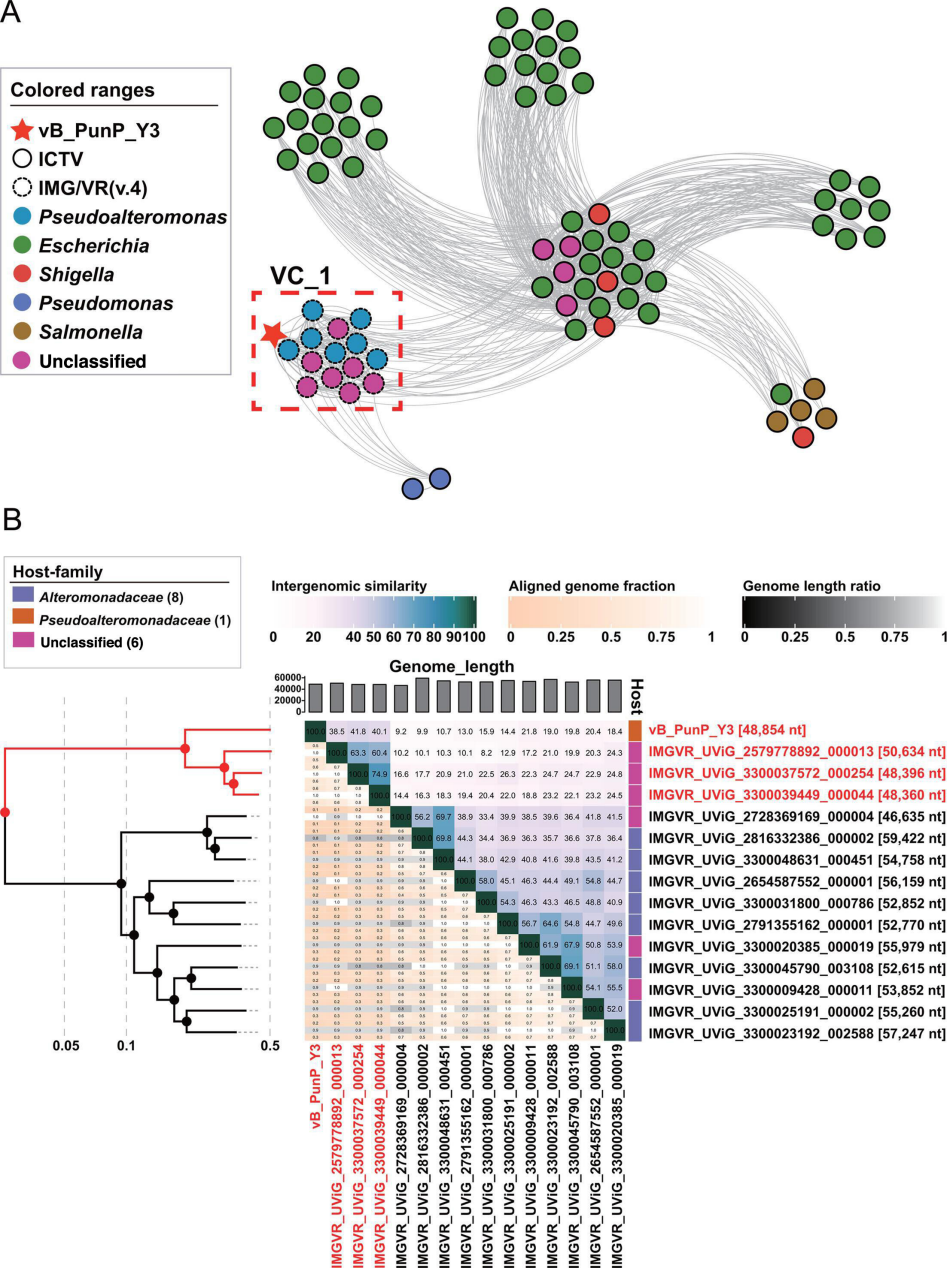
Clustering and classification of the phage vB_PunP_Y3. (A) Gene content-based viral network was created for the phage vB_PunP_Y3. The red star denotes vB_PunP_Y3. Solid lines represent ICTV data, dotted lines represent IMG/VR (v.4) data, and different fill colors indicate host bacterial genera. The red dotted box highlights viral cluster VC_1. (B) Viral phylogenetic tree and heat map of VC_1 were created based on the average nucleotide identity (ANI) values calculated using VIRIDIC.

Genome network analysis presented that 81 viruses were categorized into seven different VCs (Fig. 3A), including vB_PunP_Y3, 14 UViGs from the IMG/VR (v.4) data set, and 66 reference genomes associated with vB_PunP_Y3 from ICTV (Table S1). Among them, all UViGs along with vB_PunP_Y3 formed a specific VC, denoted as VC_1. This observation substantiates a greater protein similarity within the VC_1 group. The proteomic trees also showed the same result (Fig. S2; Fig. 3B). Additionally, the ANI analysis demonstrated that three UViGs have higher genomic similarity with vB_PunP_Y3, namely IMGVR_UViG_2579778892_000013, IMGVR_UViG_3300037572_000254, and IMGVR_UViG_3300039449_000044, and their ANI values with vB_PunP_Y3 are the highest, respectively, being 41.8%, 40.1%, and 38.5% (Fig. 3B). These findings suggest that vB_PunP_Y3 is closely related to three UViGs and may belong to a novel family. In addition, the hosts of the three UViGs were consistent with vB_PunP_Y3, suggesting that the virus of the candidate family may mainly infect *Pseudoalteromonas*.

**Fig 4:**
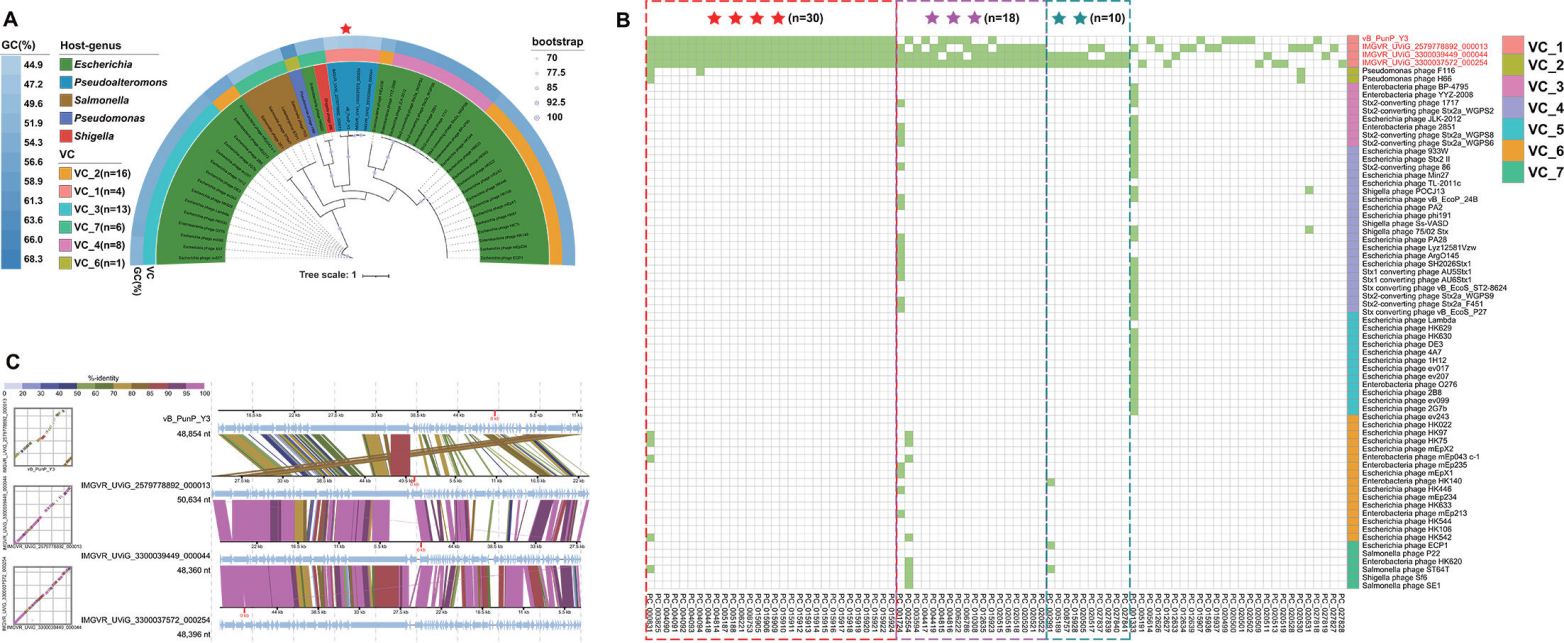
Comparative genomic analysis of phage vB_PunP_Y3. (A) Phylogenetic tree was constructed using the amino acid sequences of terminase large subunit (TerL) of vB_PunP_Y3, combined with related reference phage. Outer to inner circles represent G + C content, VCs, and host bacterial genera, respectively. (B) Homologous proteins of viruses related to vB_PunP_Y3, displayed in heatmaps. Different VCs are shown in various colors, and the number of stars within the dashed boxes indicates the number of members of *Poorviridae* with the same protein cluster (PC). (C) Genomic comparison within the *Poorviridae*, with different colors indicating genome identity.

To further confirm whether vB_PunP_Y3 and the three UViGs represent a new virus family, single-gene and whole-genome phylogenetic trees were employed to deduce the phylogenetic and genomic relationship between vB_PunP_Y3 and related reference virus. The result of phylogenetic tree for the TerL exhibited that vB_PunP_Y3 and three UViGs formed a monophyletic clade and had the most similar phylogenetic distances (Fig. 4A). These findings substantiate the establishment of a novel viral family-level cluster, including vB_PunP_Y3, IMGVR_UViG_2579778892_000013, IMGVR_UViG_3300037572_000254, and IMGVR_UViG_3300039449_000044.

Protein clustering and comparative genomic analysis are valuable tools for investigating the essential genes of viruses and establishing virus classifications (68). The outcome of homologous protein analysis unveiled that the candidate family has the most homologous proteins. (Fig. 4B). The result of genomic comparison analyses revealed that members of the candidate family exhibited high-genome similarity (Fig. 4C).

In conclusion, all the results mentioned above confirmed that vB_PunP_Y3 and the related three UViGs from the IMG/VR (v.4) data set constitute a novel unassigned viral family that may mainly infect *Pseudoalteromonas*. Moreover, vB_PunP_Y3 is the sole isolate in this putative family and the new family is designated here as *Poorviridae*.

### Comparative genomic analysis between vB_PunP_Y3 and N4-like viruses

**Fig 5:**
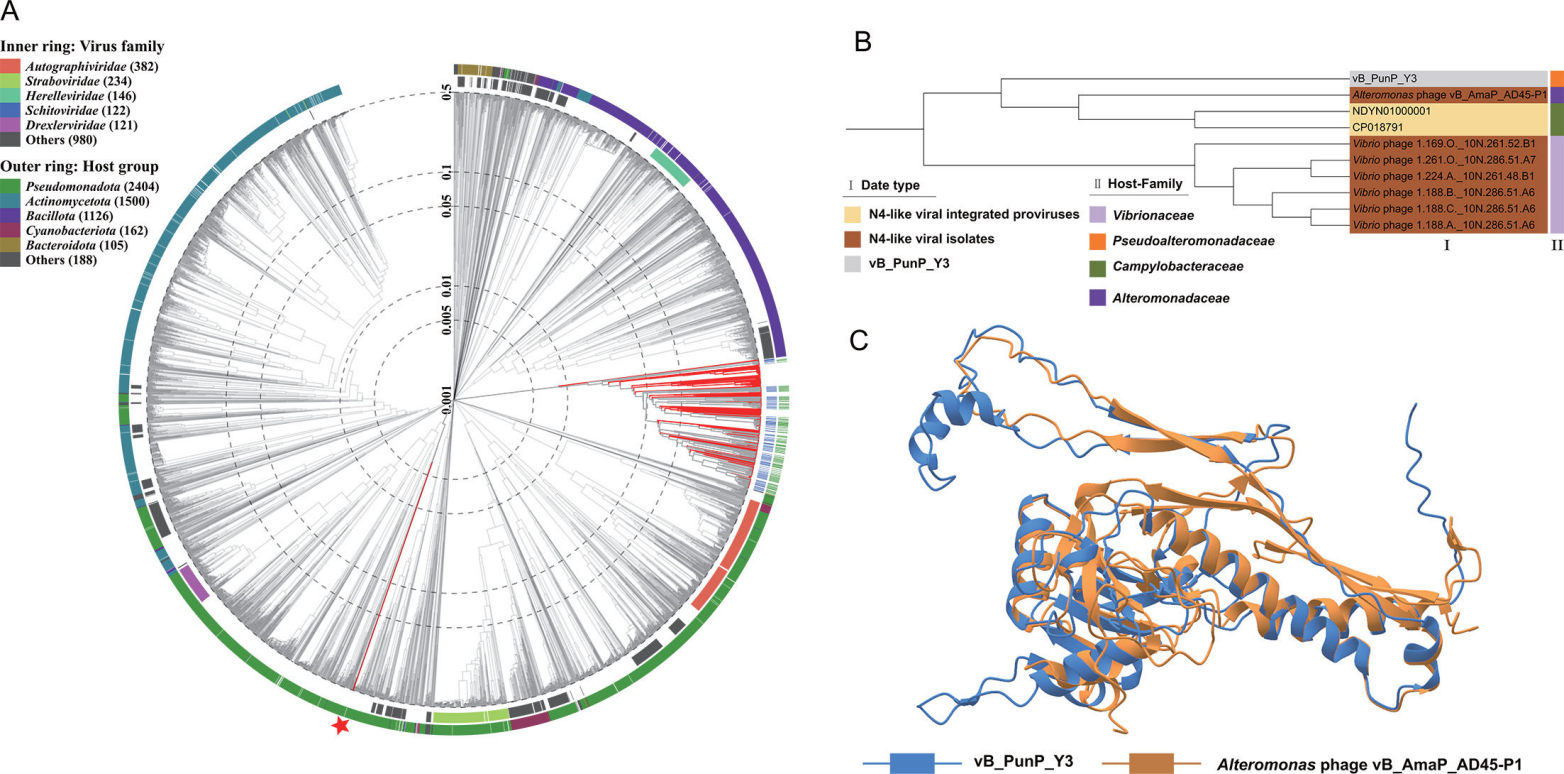
Phylogeny of phage vB_PunP_Y3. (A) Proteomic tree based on whole-genome nucleotide sequences of vB_PunP_Y3 and N4-like viruses. Outer circles show host groups, and inner circles show virus families. Red stars and solid red lines denote vB_PunP_Y3 and N4-like viruses, respectively. (B) Phylogenetic tree of the major capsid protein (MCP) from N4-like viruses and vB_PunP_Y3, with colors indicating data types and host bacterial families. (C) Comparison of the tertiary spatial structures of the MCP from *Alteromonas* phage vB_AmaP_AD45-P1 and vB_PunP_Y3.

The results of all-to-all PSI-BLAST showed that ORF13, ORF16, and ORF30 of vB_PunP_Y3 respectively, corresponded to three of the seven hallmark proteins of N4-like viruses: DNAP, MCP, and RNAP (Table S2). However, BLASTp alignment with GenBank’s nr protein database did not yield consistent results. This indicated that although there is a phylogenetic relationship between the DNAP, MCP, and RNAP of vB_PunP_Y3 and N4-like viruses, the relationship is relatively distant. The proteomic tree indicated that vB_PunP_Y3 occupied different branches compared to N4-like viruses (Fig. 5A). Additionally, the phylogenetic tree of vB_PunP_Y3 and N4-like viruses based on DNAP and RNAP also exhibited consistent results (Fig. S3). However, the phylogenetic tree of the MCP presented the opposite result that vB_PunP_Y3 was found on the same evolutionary branch with N4-like viruses (*Alteromonas* virus vB_AmaP_AD45-P1 and *Vibrio* virus 1.169.O._10N.261.52.B1), which suggested that compared to DNAP and RNAP, there is a closer evolutionary relationship between MCP of vB_PunP_Y3 and N4-like viruses (Fig. 5B). The differential selection pressures exerted on various genomic regions of vB_PunP_Y3 over an extended evolutionary period may account for this phenomenon. Additionally, N4-like viruses and the virus vB_PunP_Y3 shared a common morphology, displaying a short-tailed morphology, and the MCP of *Alteromonas* virus vB_AmaP_AD45-P1 and vB_PunP_Y3 also presented remarkably high similarity in their protein tertiary structures (Fig. 5C). Based on these findings, we hypothesized that vB_PunP_Y3 has evolved from N4-like viruses. However, various evolutionary factors, including gene mutations, horizontal gene transfer, and natural selection, have significantly altered the genome of vB_PunP_Y3, resulting in the retention of only three hallmark proteins, with the MCP exhibiting the closest evolutionary relationship. This has led to the establishment of a novel virus classification distinct from N4-like viruses, providing evidence for the emergence of viral diversity.

### Ecological distribution of *Poorviridae* in the ocean

**Fig 6:**
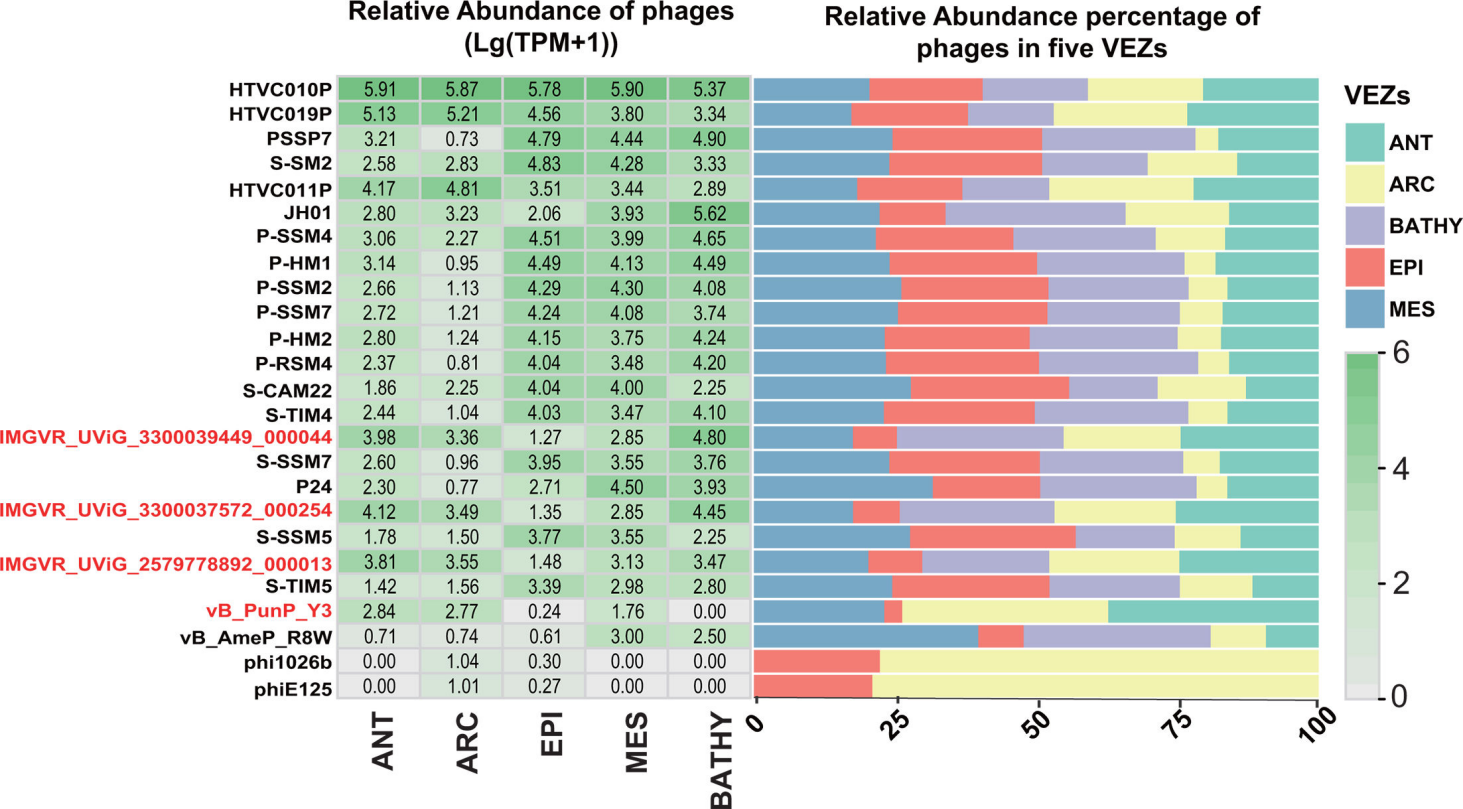
The relative abundance of *Poorviridae* family members was calculated in 154 data sets from the Global Ocean Viromes 2 (GOV 2.0) database. Phage names are listed in descending order of total abundance. Color depth represents the calculated abundance, with different colors indicating five viral ecological zones (VEZs).

The biogeographic distribution of *Poorviridae* and 21 references were assessed using data from 154 viral metagenomes sourced from the GOV2.0 database (69) (Fig. 6). The findings confirmed the high abundance of *Pelagibacter* virus HTVC011P/HTVC010P, *Prochlorococcus* virus P-SSP7/P-SSM7, and *Synechococcus* virus S-SSM7/S-SM2 across five zones (70). The virus family *Poorviridae* is widely distributed across the five VEZs. Notably, IMGVR_UViG_2579778892_000013, IMGVR_UViG_3300037572_000254, and IMGVR_UViG_3300039449_000044 are found throughout all five zones. However, vB_PunP_Y3 is primarily distributed in the ANT, ARC, and MES, with lower distribution in EPI, and no distribution in the BATHY. This difference in distribution might be attributed to the challenges of sampling in the BATHY region, where environmental complexities make it difficult to replicate conditions in the laboratory, resulting in difficulties in isolating and cultivating viruses.

### Conclusion

*Pseudoalteromonas* is a crucial member of the global marine carbon and nitrogen cycles, playing a significant role in global biogeochemical processes (71). The vB_PunP_Y3 is an isolated virus that infects *P. nigrifaciens*, and has been extensively characterized in terms of morphology, genomic characteristics, and phylogenetic features. Within its genome, three of the seven hallmark proteins of N4-like viruses were identified: DNAP, MCP, and RNAP. This discovery provides new evidence for exploring the generation of genomic diversity in viruses evolutionary. Moreover, this study revealed a novel family, *Poorviridae*, in the evolutionary lineage, which lacks cultured virus sequences, with only vB_PunP_Y3 and three UViGs from the IMG/VR (v.4) data set as its members. The study provides a detailed account of the diversity, genomic evolution, abundance, distribution details, and the unique evolutionary relationship with N4-like viruses of vB_PunP_Y3 infecting *P. nigrifaciens*. Considering the widespread distribution and crucial role of *Pseudoalteromonas* in the ocean, further research can be conducted to specifically study the isolation, diversity, and ecological roles of *Pseudoalteromonas* viruses. Additionally, our findings highlight the potential of combining metagenomics with virus isolation to enhance our understanding of the diversity and functions of marine viruses.

## Data Availability

The comprehensive genome sequence of virus vB_PunP_Y3 may be accessed within the GenBank database under accession number OR779980.

## SUPPLEMENTAL MATERIAL

Fig. S1 aem.01559-24-s0001.eps 16S rRNA phylogenetic tree of the host bacteria of the phage vB_PunP_Y3. The size of the circle represents bootstrap.

Fig. S2 aem.01559-24-s0002.eps Using nucleotide sequences of members of VC_1, a proteomic tree was generated based on the whole genome. Different colors in the outer and inner circles represent the host group and virus family, respectively. Red solid lines represent members of VC_1 and are highlighted with a red star.

Fig. S3 aem.01559-24-s0003.eps Phylogenetic trees of DNA polymerase (DNAP), major capsid protein (MCP), and virion-encapsulated RNA polymerase (RNAP) from the reference database of N4-like viruses and vB_PunP_Y3. Different fill colors represent different data types.

Table S1 aem.01559-24-s0004.xls Information for reference genomes associated with vB_PunP_Y3.

Table S2 aem.01559-24-s0005.xls Results of position-specific iterated BLAST (PSI-BLAST) between phage vB_PunP_Y3 and N4-like viruses.

ASM does not own the copyrights to Supplemental Material that may be linked to, or accessed through, an article. The authors have granted ASM a non-exclusive, world-wide license to publish the Supplemental Material files. Please contact the corresponding author directly for reuse.
